# The Impact of the Coordination between Healthcare and Educational Personnel on the Health and Inclusion of Children and Adolescents with Rare Diseases

**DOI:** 10.3390/ijerph18126538

**Published:** 2021-06-17

**Authors:** Sebastià Verger, Francisca Negre, María Fernández-Hawrylak, Berta Paz-Lourido

**Affiliations:** 1 Department of Applied Pedagogy and Psychology of Education, University of the Balearic Islands, 07122 Palma, Spain; s.verger@uib.es (S.V.); xisca.negre@uib.es (F.N.); 2 Institute of Research and Innovation in Education (IRIE), University of the Balearic Islands, 07122 Palma, Spain; 3 Department of Education Sciences, University of Burgos, 09001 Burgos, Spain; mfernandez@ubu.es; 4 Department of Nursing and Physiotherapy, Research Institute of Health Sciences (IUNICS), University of the Balearic Islands, 07122 Palma, Spain

**Keywords:** communication, interprofessional coordination, rare diseases, pediatrics, educational inclusion

## Abstract

Rare diseases produce multiple impacts for the people who suffer from them, but they also have repercussions for their families, education and healthcare. The objective of this study is to analyze the coordination between healthcare and education professionals who intervene with children and adolescents with rare diseases. It is a qualitative study designed with a critical paradigm, and it was carried out through focus group discussions. A total of 50 people participated in the study, including healthcare professionals, teachers and families. The results suggest that poor communication and coordination negatively impact minors with rare diseases, placing an extra burden on their families, who take on an intermediary role in communication. Participants in the study recognized coordination as an area for improvement as it can compromise equitable social and health services and inclusive education. Other measures must also be put into action at the public administration level not only to establish protocols for intersectoral coordination, but also to increase the knowledge and awareness of staff involved in health and education interventions for children with rare diseases.

## 1. Introduction

Rare or infrequent diseases (RDs) usually affect sufferers from birth or from a very young age and are generally chronic and without curative treatments. It is often difficult to obtain information about these conditions that would make it possible for families to cope with the disease because they are unknown or understudied diseases, and many of them do not have a specific treatment [1,2]. Although they are called RDs because each disease affects less than 5 out of every 10,000 inhabitants, there are approximately 7000 diseases called RDs, including different types of genetic diseases, some types of cancer, autoimmune diseases and congenital defects, among others [3,4]. Despite their heterogeneity, they share a number of common features. They are often severe, progressive and degenerative diseases associated with comorbidity and can cause impacts at the motor, sensory and/or cognitive level, creating a complex clinical picture, and making their diagnosis extremely difficult [5,6]. A late or nonexistent diagnosis; the absence of specific medication; and the lack of information on the characteristics of the disease, its evolution and its social–sanitary impact are therefore common obstacles encountered by affected people and their families. These challenges can also cause families difficulties in obtaining integrated care and the necessary support to meet the needs of their new reality [7,8,9,10,11,12,13,14]. Taking the multiple impacts that RDs can cause into account, the socioemotional support and advice on family management models for dealing with a child’s illness are among the challenges that health professionals have to face [15,16,17,18,19,20].

Many of these diseases appear in childhood, and therefore the schooling of children with RDs must be governed by the principles of inclusion that guide care for children in a way that considers their particular needs [21,22]. However, like other chronic diseases, RDs can have an impact not only on a child’s physical health, but also on their care, socialization and self-concept, causing difficulties in their adaptation to school [23,24,25]. Inclusive schooling at all levels depends on interdisciplinary care that is sensitive to a child´s life stage. However, it is currently recognized that RDs still pose an important coordination challenge for the scientific community and for society [26].

By contrast, the schooling of students with RDs has propelled a number of changes: first, health sector professionals (e.g., nurses, physiotherapists, occupational therapists, assistants) are carrying out their work in educational centers; second, teaching teams are responding to health problems and providing care that they had never given before; and, finally, affected families are also making a series of demands regarding the continued care of their children. Professionals have thus had to modify their expectations towards children with a disease, redefining the purpose of school so that academic achievements are not perceived as less important than for the rest of the students, while also working together with parents to set realistic goals for their children [27]. The educational experience influences the quality of life of children and requires awareness and adequate training for teachers, as well as close collaboration with families and health services [28,29,30].

This study, which is part of a larger project related to the needs of people with RDs and their families, was carried out in Mallorca (Balearic Islands), located in the western part of the Mediterranean Sea. In Spain, both the National Health Service and the Education System are tax-funded, universal for all citizens and decentralized at the regional level. The health system is organized in two main levels: primary healthcare and hospital or specialized care, added to other public and private social health services. Primary and secondary education is free for children from age 3 (preschool education) and compulsory for all children aged between 6 and 16 years old (primary education is for children aged 6 to 12 years old and secondary education is for children aged from 13 to 16 years old). As for the education of children with disabilities and chronic diseases, while there are special education schools for children with severe intellectual disabilities, in general, education legislation advocates for the inclusion of minors in ordinary schools whenever possible, as established in the Education Law February 2006 [31] and in the recently approved Amendment to the Law March 2020 [32]. Sometimes, children who are ill for a prolonged period have the possibility of being home-schooled through a special service of home support teachers provided by the education administration. At the school, the teacher is responsible for all the students that make up his or her class, but they may have the help of other professionals such as support teachers for students with specific needs or hearing and language teachers who provide special attention to students with hearing and/or language development problems. In addition, there are special education assistants (ATEs in their Spanish initials), who provide assistance in activities of daily life that students with special educational needs cannot do independently, always under the responsibility of the teaching staff.

Taking into account that children with RDs receive health and educational interventions and, at the same time, that RDs are largely unknown, the aim of this study is to analyze communication and coordination between healthcare providers and teachers from the perspective of families and health and education professionals.

## 2. Methodology

This is a qualitative research study with a social–critical paradigm. This paradigm incorporates many different perspectives [33], but what is common among them is that the research must be sensitive to the needs of vulnerable groups in society and also provide practical recommendations to avoid the injustices that underlie everyday actions. Specifically, the critical approach of Paulo Freire [34] not only allows for a transformative review of the data, but the research process itself is articulated in a way that builds awareness of the research topic among participants. It also takes into account the theoretical transition that disability studies have undergone in recent decades up to the current focus on functional diversity [35]. In this sense, minors with RDs and their families require equitable interventions that are adjusted to their particular needs based on the social impacts of the disease; by identifying deficiencies in communication and coordination between the different public sectors we may not only avoid overlaps, delays and adjustment in the care received by minors, but also improve the quality of life of families.

### 2.1. Eligibility and Sample Recruitment

Participants were contacted through key informants, with the collaboration of schools and the Balearic Association of Families of Children with Rare Diseases (ABAIMAR in its Spanish initials), which includes close to 100 families. Through these institutions, participants volunteered to take part, including primary and secondary education teachers from public schools, health workers from pediatric services and ABAIMAR member families. The sample consisted of 50 participants, divided into the following profiles: 8 parents of children with RDs (6 women and 2 men), 3 nurses (women), 5 school physiotherapists (3 women and 2 men), 8 special education assistants (women), 6 primary school teachers (4 women and 2 men), 6 primary school assistant teachers (5 women and 1 man), 3 hearing and language teachers (women), 2 primary school educational psychologists (1 man and 1 woman), 3 secondary school teachers (2 women and 1 man), 4 secondary school teachers (2 women and 2 men) and 2 secondary school educational psychologists (women). As for parents, families with children with different rare physical and cognitive disorders attending primary (4) or secondary education (5) were selected. As for teachers, the selection criteria for all teaching professionals implied prior experience with children or young people with RDs, with a minimum of one academic year. Participants’ ages ranged from 32 to 56 years old.

### 2.2. Data Collection Instruments

Information was obtained through in-depth, semi-structured interviews conducted through 6 focus groups that were made up of professionals and families with different profiles (Table 1). Based on the understanding that groups that are too heterogeneous can hinder group dynamics when research topics may be sensitive for participants [36], we opted to group participants according to their professional profile, or the education level they work in, while families were included in a separate focus group. In this manner, each group could express their opinions freely and confidentially.

The teaching staff was divided into four focus groups. Two similar groups were made up of teachers and teaching assistants from preschools and primary schools; another group consisted of staff from the guidance team, a support teacher, and a hearing and language teacher from secondary school; and a final group was formed by special education assistants from both primary and secondary schools. The healthcare workers’ focus group included physiotherapy staff who perform interventions in schools and nurses from primary healthcare centers. The final focus group was made up of parents of students with RDs.

The focus group discussions were held in a quiet room located at the University of the Balearic Islands. On average, they lasted for 90 min, the criteria for ending them being when a saturation of information had been reached. Two people from the research team participated in each session, one acting as moderator and the other as observer. Two digital recorders were used to record the session. The sessions were then transcribed carefully considering confidentiality issues; as such, members of each focus group were identified with the initials FG and a number–letter combination where the number referenced the focus group and the letter identified the individual participant within the group.

### 2.3. Data Analysis

A discourse analysis was carried out by two researchers (women) and a researcher (man) with different academic profiles in the education and health fields. They began by reading the transcript and ordering and classifying the information by units of discourse analysis. The contributions of the different focus groups were contrasted with data from other phases of the study developed between 2016 and 2020, in order to ensure triangulation of the information and guarantee methodological rigor [37]. Previous phases of the study included an extensive literature review and in-depth interviews and focus groups with children, experts in the field and key informants [38]. Researchers were members of different research groups in the area of health and education, and transcription was developed by an independent research assistant with an academic background in psychology and previous training in qualitative research. Ethical issues were considered both in the development of the group interviews and during the analytical process. Confidentiality was assured in all research phases. The project was approved by the research ethics committee of the University of the Balearic Islands.

## 3. Results

The results of this study highlight that coordination between schools and health centers is scarce; that it is not always carried out with protocols; and that there are barriers, but also opportunities for an improvement in the future that will benefit families, children and the equity of the healthcare and education system. This section groups the different categories that influence current coordination and the aspects related to improved coordination in the future. These nine categories refer to intersectoral coordination, knowledge about rare diseases, healthcare providers, communication with families, communication with students, interprofessional collaboration, specific support, school size and protocols for communication. These categories show different areas of intervention to improve coordination and communication.

### 3.1. Intersectoral Coordination

Intersectoral coordination between schools and health services is scarce and fundamentally related to health emergencies, since schools must have an established intervention protocol that is known to teachers:

*“If we have to intervene, we already know which specific injection to administer, that it is in Management, that we have to notify the head of studies, that he will notify the family, that they will take [the child] to the doctor, etc.”*.

(FG_2b)

Focus group participants consider that the coordinated action protocol that is put into effect when a health emergency occurs in the school is insufficient, no matter what case, highlighting the need for continued coordination not only for children with RDs, but for the benefit of all students. Primary school teachers, in particular, show this need in the following quote:

*“…yes, there ought to be coordination in between schools and health services, not only in relation to boys and girls with RD, but sometimes we also need information on what the health protocols are in general”*.

(FG_1Af)

In addition to the advantages for the areas of healthcare and education, coordination between these sectors is valued as a way to improve the relationship with the families of children with RDs, since they would feel more informed and supported by both sectors:

*“It would be a very appropriate measure since it would enable the most efficient and highest quality treatment for students with RD, as well as the feeling of security and support for families”*.

(FG_1Ah)

Healthcare workers consider that coordination in preschools and primary schools is easier than in secondary schools where the teaching staff is larger and it is not as easy to have a reference person who is in daily contact with the children:

*“There is a difference between a primary school and a secondary school. In primary school, you have a main teacher, a reference person. In secondary school too, but this figure is fuzzier”*.

(FG_4d)

They view an improvement in the coordination between institutions and professionals as an opportunity that will encourage a better adaptation to the educational needs of children and their healthcare, while also relieving the families of all the bureaucratic procedures they are subjected to. For this group, there is still a lack of awareness of the public administration:

*“We only ask that they understand their situation, and that they try to adapt to their needs. Nothing more”*.

(FG_5i)

### 3.2. Knowledge about Rare Diseases

In the opinion of the families, the root of the problem of the lack of coordination between different sectors of the public administration lies in the great ignorance that still exists about RDs and their implications for families. They face a series of disadvantages such as barriers to obtaining economic aid and the problem of financing medication or other essential technical aids, in comparison to other chronic diseases.

*“It’s a very expensive disease. Social Security does not cover anything for me”*.

(FG_5h)

This leads families to feel discriminated against compared with other families whose children are not sick, or those that have more well-known diseases. They demand understanding, communication and coordination so that their children can have equal opportunities like the rest of the students:

*“Above all, understanding is needed. Everybody should understand that the situation is not easy, especially for the children”*.

(FG_5g)

In addition, if the child’s disease does not appear on the list of known diseases, as is the case for many RDs, families will not be able to ensure the advantage of choosing a school that is being offered to other children with highly prevalent chronic diseases:

*“When you enroll a child in a school there are a number of diseases that give you extra points to secure a place. But in this entire list, there aren’t any rare diseases!”*.

(FG_5g)

### 3.3. The Role of Healthcare Providers

Regarding the role of health professionals in giving support to schools, participants noted that the nursing staff of the primary healthcare centers sporadically conduct talks, in addition to other educational activities on health promotion. During these talks, they explain what happens when a child with a particular disease has a health crisis and how to intervene and administer medication. However, there is agreement among the teaching staff that better coordination with health centers could ensure continued care for minors and safety at schools.

*“Yes, I think that this coordination would be very necessary to be able to find a solution to all those doubts that arise on a day to day basis, and to be able to carry out an intervention as effectively as possible”*.

(FG_1Ai)

According to nursing staff in primary healthcare centers, it is important to decentralize the activities of the hospital, since continuous care can be carried out from the healthcare centers. In the case of physiotherapy, physiotherapists are hired by the school administration to promote attention to diversity and inclusion, and it is considered a highly valued resource. Thus, it is clear that minors can be in contact with health professionals of different levels according to their situation, while reserving specialized care for those moments when it is necessary, which will require specific communication with hospital staff:

*“…the children are perfectly controlled, and need to be hospitalized less”*.

(FG_4f)

Physiotherapy interventions take place in the school in order to centralize educational and health interventions in the same space. However, that requires coordination to adjust physiotherapy sessions to school programming.

*“The physiotherapist needs a lot of coordination with the head of studies to balance the schedules of attention to the students”*.

(FG_4e)

### 3.4. Communication with Families

Teachers point out the importance of communication with families as they are the priority source of information on the situation of their children. However, they note that their relationship with families can vary, as can the kind of involvement and the information available to the teaching community.

*“Coordination with healthcare workers is practically nonexistent. Medical information comes to us through the family via supporting documents or medical documents”*.

(FG_1Ai)

*“Whether there is more or less involvement depends on the family, which is based on how they assimilate the disease”*.

(FG_1Ad)

*“The most serious problem (…) is when parents do not worry about when there is a change in medication or of another type, and do not communicate it to the school. If they don´t, you have to pursue them asking what happened to the child”*.

(FG_2b)

This information is very important for teachers since there are cases of students with RDs who go unnoticed because their disease is not visible. In such cases, they will not receive educational attention adapted to their needs nor will it be identified if a health problem arises:

*“…it is necessary for teachers to be informed as there are cases of students with rare diseases that go unnoticed”*.

(FG_2a)

Thus, families have a fundamental role not only in the transfer of information between professionals in the health and educational sectors, but also in training and sensitizing teachers to their child’s problems. This can become an added burden on families.

### 3.5. Communication with Students

The teachers´ focus group indicates that the attitudes of families may be different in preschool and primary than in secondary school. Older students may have a greater capacity for self-management of their disease, so families no longer communicate to the schools that their child has a chronic disease.

*“The student already knows their illness, and knows how to take their medication, so families don´t inform schools that they have a chronic disease, about which they should inform the school”*.

(FG-2b)

*“You see a kid with an inhaler, for example, in their backpack. You ask them, what is this?”*.

(FG_2c)

In terms of follow-up at the school, teachers state that when students are older, they know about and manage their disease on their own, and they do not usually communicate their pathology to the school, making it difficult to include them in the school´s protocol.

*“They know their disease, and perceive when they will have a crisis. They know how to administer the treatment themselves. It’s not that they notify us. They take their medication by themselves”*.

(FG_2c)

### 3.6. Interprofessional Collaboration

There appears to be greater communication and coordination between different groups within the education sector, or with families, than with healthcare staff. These quotes highlight the general perception:

*“Coordination with healthcare staff, none. With the families, continuously”*.

(FG_1Ab)

*“Direct coordination with healthcare staff is nil”*.

(FG_2Af)

In general, teaching staff consider it necessary to improve coordination with healthcare professionals. They have doubts about what to do to facilitate the day-to-day with the child in class, or at specific moments of greater complexity, and recognize that they are unaware of certain procedures and the consequences for the children:

*“I didn´t know how to act in some situations, and I would have liked more coordination with the medical staff”*.

(FG_1Ac)

Even so, specific experiences of continuous communication and coordination between the staff of primary schools and specialized healthcare centers were described, which contributes to the perception there is a possible margin for improvement.

*“We have a diabetic child who is 3 years old… we speak twice a week with the hospital nurse”*.

(FG_4b)

Regarding coordination among education professionals, the teacher is considered key, since he or she will be the one who will detect a child´s situation. However, the involvement of the school management and the guidance department is required. In addition, they understand that the teacher is key in activating the entire team:

*“The guidance team will find out what resources exist in the community, make contact, etc. The teacher doesn´t do that, but it’s the teacher who has detected the need, and has communicated this to the guidance department. The guidance team, the teacher and the parents then make the decision”*.

(FG_2e)

Healthcare staff also revealed that communication and coordination depend on schools in general and on the attitude of the child’s guardian in particular.

### 3.7. Specific Support

The role of support staff, such as ATEs, was also emphasized. It is seen as fundamental in the quality of life of children and as a support for both intersectoral communication and communication with families. For this reason, teachers refer to the need to speed up the coordination between the schools and school administrations to cover the ATEs’ short-term absences or days off and avoid situations that generate exclusion for minors:

*“…many times, when an ATE does not go work, the family is advised by the school or the Department of Education that the child should not go to school”*.

(GF_3a)

In addition, evidence suggests that it is fundamental that all support persons who intervene with children, in particular the ATEs, have clearly determined functions. They should carry out their work according to what is established in school practice to avoid conflicts or unsafe situations, for example when caring for or administering medications to minors:

*“On paper, there are responsibilities established by collective agreement, with instructions from the Department of Education of what we have to do. What happens is that you end up doing much more”*.

(FG_3e)

*“These are interventions with young children, we provide support but we are not healthcare workers”*.

(FG_3e)

### 3.8. School Size

In general, the discussions point to a lack of pre-established and agile protocols among education and health professionals and families. However, in small schools, the transfer of information can be carried out more easily due to the relationships established between the teachers in management or faculty meetings, through medical or educational psychology reports, conversations and formal or informal exchanges with the families:

*“Because of the family environment and the daily contact with all the teachers, information is passed orally and through the reports”*.

(FG_1Aa)

In secondary schools, the management team is the one who has the information about the protocol to follow, not the school counselors. In any case, this means that:

*“The transfer of information is complicated, above all in large schools that have a lot of students”*.

(FG_2a)

### 3.9. Establishing Protocols for Ongoing Communication

Finally, although there is a convergence in all the teachers’ focus groups regarding the need to have coordination protocols with the healthcare team, the lack of resources, means and institutional will makes this seem like a utopia:

*“I believe there should be an established protocol on coordination between the schools and healthcare services. This would be very positive, but it would be very difficult to make this a reality”*.

(FG_1Bd)

Focus groups considered that the responsibilities for coordination should lie with the school´s management team and the guidance department. Another proposal is to improve coordination through direct communication with the healthcare services who care for the minor, without intermediaries:

*“There should be a direct connection between hospitals and continual care”*.

(FG_4f)

## 4. Discussion

The results of this study show various points of coincidence between the different focus groups, such as the need for coordination not only between health and educational professionals, but also between professionals from the same group, with the participation of families and public administrations included in the process as well. They demonstrate that, in many cases, coordination is based on the willingness of the groups to communicate. The transfer of information also depends on the collaboration of families, who often only get continuous care adapted to the needs of the child by insisting on it.

Teachers state the need to have information on health concerns in order to make personalized adaptations. At the same time, the participation and coordination of the teaching team emerge as the essential conditions to achieve real inclusion. All this suggests the need for networking that includes two-way communication between schools and hospitals, while also considering community structures such as the primary healthcare centers. Both groups must participate in improving care within the educational setting, with the understanding that it is crucial to improving the quality of life of patients with RDs.

Teachers who have students with RDs in the classroom can have fears such as not having the information or training necessary for them to offer an adequate and quality response. Transdisciplinary training should be required for teachers to enable them to make decisions or carry out interventions that go beyond their professional profile, regardless of the need for better collaboration with healthcare professionals. Additionally, a social, health and educational space, that also includes families, should be formed where common goals and agreed criteria can be shared, to enable the inclusion of children with RDs in a school setting and ensure that families feel protected from the consequences of the disease and accompanied in the procedures required by the administration.

The role of other community services and members of the third sector, such as associations, is critical to facilitating this coordination. In addition to organizing events to raise awareness of rare diseases and representing families before the administration, they provide parents with a forum to share experiences that could be useful in pushing for the inclusion of their children. These organizations are a loudspeaker for the advances in research and policy in this area at the local, national and international levels. Families and staff at schools and healthcare services can contact patient advocacy groups such as FEDER, EURORDIS, ALIBER, Orphanet Database or the Genetic and Rare Diseases Information Center to develop solutions tailored to the needs of children with RDs and their families.

Regarding the protocol for the continuous transfer of information, focus groups confirmed yet again the lack of previously defined procedures, as called for in the literature [39]. In the context studied, informal conversations appear to be the most common mechanism for transferring information among the teaching staff, although faculty meetings or other meetings are also used. Medical and educational psychology reports are usually the most used instrument for knowledge transfer. However, the main means of communication and coordination are the families with whom communication is established informally at the beginning or end of classes, or through a specific meeting. In this study, coordination with secondary schools was perceived as more complex than in preschool and primary education, where it is easier to identify reference teachers. This also affects families, since the further their children advance in their studies, the more teachers intervene in their education, necessitating communication with a variety of teachers.

The relationship with the family is essential in managing the educational intervention adequately. However, there are different family models, and the level of difficulty in coordinating depends on what the family is like, how they experience the disease and the information they have at their disposal to deal with the disease. It is important to mention the repercussions of these types of diseases on those affected and their families, such as the difficulties in obtaining a diagnosis, the difficulties in coping with symptoms, a lack of information about the disease, difficulty in obtaining healthcare or financial resources, a lack of availability of drugs and the experience of disability and personal impact, as well as the consequences on the quality of family life as a whole.

Participants in the study consider that coordination with healthcare staff is insufficient, although they perceive it as essential to be able to offer quality educational care. This highlights the challenges that still exist [40,41] in order to have good communication between hospitals and schools and to facilitate the exchange of information. Coordination does not only refer to the exchange of information, documentation and recommendations. It is also essential when designing class schedules and physiotherapy sessions or when agreeing on resources that may be needed, such as furniture, technical aids, food and the caretaking of the child with RD, among other relevant aspects. All of this leads to the need for both the health and educational systems to become more aware of the situation of children with rare diseases in order to understand why greater coordination and communication is so necessary. A standardized education that is not sufficiently sensitive to their needs, even when there lacks a clear diagnosis, can place these students in a position of vulnerability and distress in relation to their peers. Health care that does not take into account the educational and social role of the school and has a narrow focus on therapeutic interventions, does not consider health equity as a whole. 

Different studies highlight the importance of starting training on RDs right from university [41,42,43,44,45,46] so that professionals from different disciplines have a real and nonstereotyped vision of these types of afflictions and their implications. Besides this, training in skills such as communication and coordination should also be reinforced, while requiring the implementation of intersectoral education programs. Additionally, it is necessary to clearly identify the competencies and functions of the support staff, in particular of the ATEs. Their responsibilities are not adequately defined, and what is being demanded of them does not necessarily correspond with their qualifications or conditions in their contract, a situation that sometimes creates conflict with families and teachers.

Identifying social–sanitary needs is a fundamental step in organizing the care system and in order to improve coordination with teaching teams. However, it is also necessary to identify the role that children and families play, in order to detect situations of vulnerability or extra burdens on families. The results of the study show that, according to the perception of the participants, it is possible to improve the care offered when there is good communication and coordination between health and educational professionals, with benefits not only for children but also for their families and for the professionals themselves. For this, a protocol must be established that is shared by the different stakeholders and that harmonizers the transfer of information and the coordination. It should have key reference staff members in place in each sector for each particular situation, ensuring that the necessary training, procedural and technological solutions are available to them that will enable this exchange. Greater effort is needed to transform the plans and programs described in social and health public policies [47] into an effective practice with an impact on children and their families, reinforcing the role of the structures created to facilitate coordination [48,49].

This study has several limitations that should be considered. In the first place, and due to the very nature of a study on rare diseases, the experience of families, teachers and health professionals can be influenced by the specific characteristics of a child´s illness and its social health impact. The specific diagnoses have not been taken into account in the selection of the participants, although an attempt has been made to capture variability in the type of affliction and level of required support. Setting up heterogeneous groups with families, teachers and health workers could have been useful for the development of future coordination measures, as well as to identify the associated problems for their implementation. This study is contextualized but has the potential to be transferred to similar contexts. However, there is a diversity in the management of coordination and the types of professionals who intervene with children with rare diseases, so it is necessary to carry out other studies to understand how intersectoral coordination occurs in other regions where the presence of healthcare figures (e.g., nurse school, physiotherapist) or educational specialists (e.g., hearing and language) is scarce or nonexistent, in order to design strategies that can be implemented at the state level, with a goal of sustainability. Future studies could examine the role played by the primary healthcare centers, since this level of healthcare incorporates a community and family aspect that could be particularly useful in the case of children with RDs.

## 5. Conclusions

This study emphasizes the difficulties for equitable schooling for children with RDs, illustrating different challenges for education and health sectors, with benefits for children and their families.

The results confirmed the need for an improvement in intersectoral coordination to both facilitate and improve the professional practice of teachers and health workers, as well as to enable greater support for families in order to avoid them playing an intermediary role. Although the relationship with preschools and primary schools may be made easier by the smaller number of teachers who intervene with the children, it is not without challenges and aspects that demand changes, such as the lack of knowledge about RDs and their repercussions. This also includes the perception of families that feel discriminated against, uninformed and neglected, as their needs are multidimensional and require the coordination of the different public sectors.

Therefore, a multidimensional intervention is necessary that affects not only the educational and health administration, but also the teaching community, the professionals who intervene in the schools and the support staff. Families and children themselves must be listened to and understood, since they are the group that participates in both health and educational spaces. The use of communication technologies can facilitate the needed coordination because, although some factors such as the size of the school cannot be changed, they enable the provision of more individualized attention adapted to the needs of the children with RDs so that the educational requirements do not compromise their health and that during periods of absence from school they can maintain the socialization and education provided by the school.

Finally, the results of this research help to advance an understanding of inclusive processes that ensure the right to education and comprehensive health of students with RDs, but more research in this area is needed.

## Figures and Tables

**Table 1:** Sample and participants’ distribution in focus groups.

	Number of Focus Groups: 6				
Sector	Primary and Secondary Schools			Health Institutions	Families
FG Identification	GD_1A/GD-1B	GD_2	GD_3	GD_4	GD_5
Profile	Teachers Tutors Assistant teachers ATEs	Guidance and counseling school personnel Assistant teachers	ATEs Teachers	Nurses Physiotherapists	Parents
Total	10 and 10	5	6	10	9
Participant Identification	a…j/k…s	a…e	a…f	a…j	a…i

## Data Availability

The data are not publicly available to ensure the anonymity of the participants.
